# *Toxoplasma gondii* Is Dependent on Glutamine and Alters Migratory Profile of Infected Host Bone Marrow Derived Immune Cells through SNAT2 and CXCR4 Pathways

**DOI:** 10.1371/journal.pone.0109803

**Published:** 2014-10-09

**Authors:** I-Ping Lee, Andrew K. Evans, Cissy Yang, Melissa G. Works, Vineet Kumar, Zurine De Miguel, Nathan C. Manley, Robert M. Sapolsky

**Affiliations:** 1 Department of Biology, Stanford University, Stanford, California, United States of America; 2 School of Biological Sciences, Nanyang Technological University, Singapore, Republic of Singapore; 3 Department of Neurosurgery, Stanford University School of Medicine, Stanford, California, United States of America; 4 Stanford Stroke Center and Stanford Institute for Neuro-Innovation and Translational Neurosciences, Stanford University School of Medicine, Stanford, California, United States of America; 5 Department of Neurology and Neurological Sciences, Stanford University School of Medicine, Stanford, California, United States of America; Stanford University, United States of America

## Abstract

The obligate intracellular parasite, *Toxoplasma gondii*, disseminates through its host inside infected immune cells. We hypothesize that parasite nutrient requirements lead to manipulation of migratory properties of the immune cell. We demonstrate that 1) *T. gondii* relies on glutamine for optimal infection, replication and viability, and 2) *T. gondii*-infected bone marrow-derived dendritic cells (DCs) display both “hypermotility” and “enhanced migration” to an elevated glutamine gradient *in vitro*. We show that glutamine uptake by the sodium-dependent neutral amino acid transporter 2 (SNAT2) is required for this enhanced migration. SNAT2 transport of glutamine is also a significant factor in the induction of migration by the small cytokine stromal cell-derived factor-1 (SDF-1) in uninfected DCs. Blocking both SNAT2 and C-X-C chemokine receptor 4 (CXCR4; the unique receptor for SDF-1) blocks hypermotility and the enhanced migration in *T. gondii*-infected DCs. Changes in host cell protein expression following *T. gondii* infection may explain the altered migratory phenotype; we observed an increase of CD80 and unchanged protein level of CXCR4 in both *T. gondii*-infected and lipopolysaccharide (LPS)-stimulated DCs. However, unlike activated DCs, SNAT2 expression in the cytosol of infected cells was also unchanged. Thus, our results suggest an important role of glutamine transport via SNAT2 in immune cell migration and a possible interaction between SNAT2 and CXCR4, by which *T. gondii* manipulates host cell motility.

## Introduction

*Toxoplasma gondii*, an obligate intracellular protozoan that is capable of infecting nearly all warm-blooded animals, depends on the host to meet its glucose and energy requirements. Recent studies have shown that apicomplexan parasites catabolize both glucose and the non-essential amino acid glutamine via the tricarboxylic acid (TCA) cycle to generate energy; however, disrupting the entry of glucose-derived intermediates into the TCA cycle has no significant effect on the growth of asexual stages of *Plasmodium falciparum*, indicating that glutamine serves as an alternative carbon source for the cycle [1]. *T. gondii* has also been shown to rely on glutamine for energy during glucose starvation, and glutaminolysis is indispensable to its survival [2]–[4]. Rapidly dividing tachyzoites of *T. gondii* may therefore impose a heavy glutamine burden on the host, and we here propose that such glutamine dependency may generate a specific means by which the parasite can manipulate the function of host cells to induce them to leave the circulation and enter tissues with high glutamine level for the benefit of the parasite. *T. gondii* has been shown to migrate to and encyst in brain, retina and muscle during chronic infection, all places with abundant glutamine supply [5]–[9].

*T. gondii* has been demonstrated to manipulate the migratory properties of infected immune cells, inducing a hypermotility state in infected dendritic cells (DCs), which has been hypothesized to promote dissemination of parasites throughout the body [10], [11]. This phenomenon is dependent on G*i*-protein signaling but the specific *Gi* protein-coupled receptor has not been identified. C-X-C chemokine receptor 4 (CXCR4), the unique receptor for small cytokine stromal cell-derived factor-1 (SDF-1) on the surface of immune cells, may be a strong candidate. The SDF-1-CXCR4 complex activates the downstream phosphoinositide 3-kinase (PI3K) pathway involved in cell motility [12], [13]. Importantly, the PI3K pathway can also be induced by the glutamine transporter (sodium-dependent neutral amino acid transporter 2, SNAT2)/amino acid substrate complex in muscle cells [14], [15].

SNAT2, the ubiquitously expressed subtype of the system A transporter in mammalian cells, is the primary mediator of uptake of aliphatic amino acids such as glutamine, glycine, and asparagine [16]. Recent studies on cultured rat myoblasts have demonstrated that SNAT2 exhibits a hybrid transporter-receptor (transceptor) function that can sense extracellular amino acid concentration and activate downstream PI3K pathway during nutrient stress [14], [15]. Such amino acid transceptors are well documented in *Drosophila* and yeast like *Saccharomyces cerevisiae*, but the role of SNAT2 in mammalian immune cells has not been extensively studied. On the other hand, it is well known that lymphocytes, monocytes and macrophages have high utilization rates of glutamine, which is involved in cell proliferation, expression of surface activation markers and the production of cytokines [17], [18].

In this study we hypothesized that *T. gondii*, due to its high demand for glutamine, manipulates the dual transporter/receptor function of SNAT2 in the host, and via interaction with the CXCR4 signaling pathway causes enhanced migration of infected immune cells to glutamine-enriched environments. In order to determine the specific role of glutamine and the transporter SNAT2 as variables influencing this migration, we used an *in vitro* transwell system to investigate cell migration of normal and *T. gondii*-infected murine bone marrow-derived DCs to a range of L-glutamine concentrations. We examined the role of SNAT2 and CXCR4-PI3K-Rho kinase pathways in this migration. Our data show that CXCR4 signaling-dependent migration is SNAT2-dependent in normal DCs, and antagonizing both CXCR4 and SNAT2 blocks the induced migration in *T. gondii*-infected cells.

## Results

### *T. gondii* is dependent on glutamine for optimal infection, replication and viability *in vitro*

**Figure 1:**
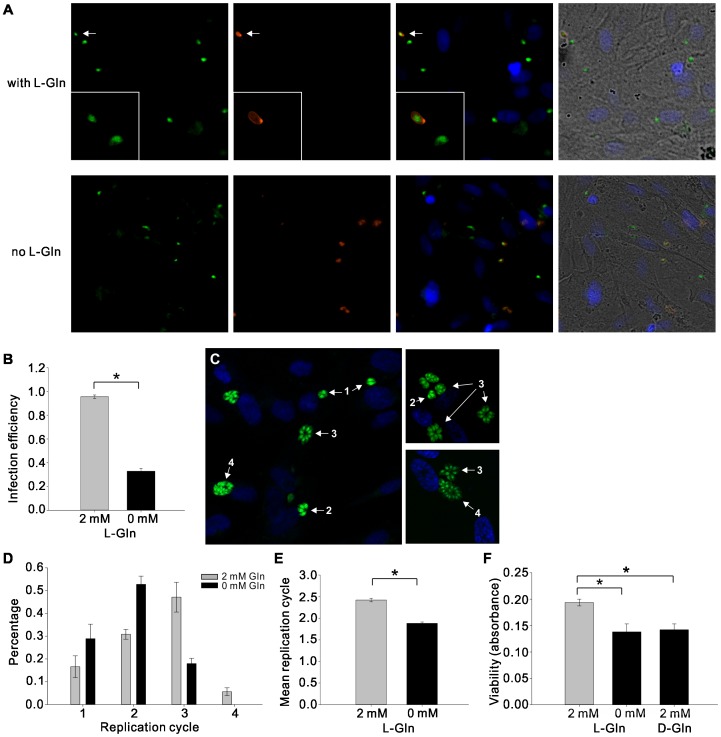
*T. gondii* is dependent on glutamine for optimal infection, replication and viability *in vitro*. Experiments were conducted in human foreskin fibroblast (HFF) monolayers. (A) Representative confocal images on the top row show the intracellular tachyzoites expressing GFP (green) and one extracellular parasite staining with SAG1 (red, also indicated by the white arrow) in the presence of L-glutamine. A 63× oil objective was used to examine the SAG1-positive parasite at higher magnification (bottom left corner). Pictures on the second row represent the SAG1 staining of extracellular *T. gondii* in the absence of L-glutamine. The nuclei of HFFs were stained with DAPI (blue), and the merged images of the fluorescence and the bright field shows that the HFF monolayer was confluent when the experiment was carried out. (B) Infection efficiency is defined as the ratio of intracellular parasites to total parasites in the examined field on the coverslip after 6 hours of infection. Infection efficiency was reduced ∼65% by omitting L-glutamine (Student's t-test, P<0.001). (C) Examples of the confocal images showing *T. gondii* replication in HFFs after 22 hours in the presence of glutamine. The replication cycle numbers were indicated in white. (D) In the presence of L-glutamine, *T. gondii* tachyzoites were able to duplicate up to 4 times in 22 hours, but without L-glutamine, over 50% of parasites only divided twice. (E) In the absence of L-glutamine, mean replication cycle number declined approximately 25% in 22 hours (Student's t-test, P<0.05). (F) MTT assay was used to evaluate parasite viability. Glutamine starvation significantly decreased the viability of *T. gondii* by 29%, and D-glutamine was unable to substitute for L-glutamine (one-way ANOVA, F_(2,89)_ = 45.036, P<0.001). Asterisks indicate significant difference from medium containing 2 mM L-glutamine (post hoc Dunnett's test, P<0.001). Data show mean value ± SEM from three independent experiments.

Type II strains of *T. gondii* (e.g., Prugniaud) are the most prevalent in Europe and North America [19], [20], are less virulent in the mouse model but highly associated with human diseases [21], [22], and they alter host physiology and behavior [23], [24]. Therefore, we first investigated if type II tachyzoites maintained in human foreskin fibroblast (HFFs) monolayers depend on glutamine for infection, replication, and viability *in vitro*. INFECTION: By using live staining of SAG1 (red), the major surface antigen of *T. gondii*, we were able to distinguish extra- (red and green) and intracellular (green) tachyzoites and quantify the efficiency in infecting HFFs (Fig. 1A). In the absence of glutamine, no obvious change of the morphology of parasites was observed. However, without glutamine, there were more extracellular parasites in the examined fields of the coverslips over the course of 6 hours and the average of infection efficiency of *T. gondii* was reduced approximately two-thirds (Fig. 1B). REPLICATION: HFF monolayers were firstly infected with *T. gondii* tachyzoites in the presence of glutamine for 6 hours. Extracellular parasites were then washed away with phosphate-buffered saline (PBS) and infected HFFs were cultured in fresh medium containing 0 mM or 2 mM of L-glutamine for another 16 hours. In the presence of glutamine, *T. gondii* was able to duplicate up to 4 times in 22 hours (Fig. 1C) while the replication ability was impaired with ∼50% of the intracellular tachyzoites only having divided twice after glutamine deprivation (Fig. 1D). The mean number of replication cycles was quantified and figure 1E shows that the replication ability of *T. gondii* was reduced about 25% in the absence of glutamine. VIABILITY: Parasite viability was evaluated by a tetrazolium dye reduction (MTT) assay, and after 4 hours of glutamine starvation, the viability of *T. gondii* decreased 29% and could not be rescued by the stereoisomer D-glutamine, which suggests that glutamine serves as an energy source for *T. gondii* metabolic activity rather than a modulator of extracellular redox environments (Fig. 1F). MTT assays showed no significant effect of L-glutamine depletion on the HFF host cells (Fig. S1), indicating that the virulence and growth of *T. gondii* are not compromised by the host viability during glutamine starvation.

### Infected DCs exhibit hypermotility and an enhanced migration specific to high glutamine *in vitro*

**Figure 2:**
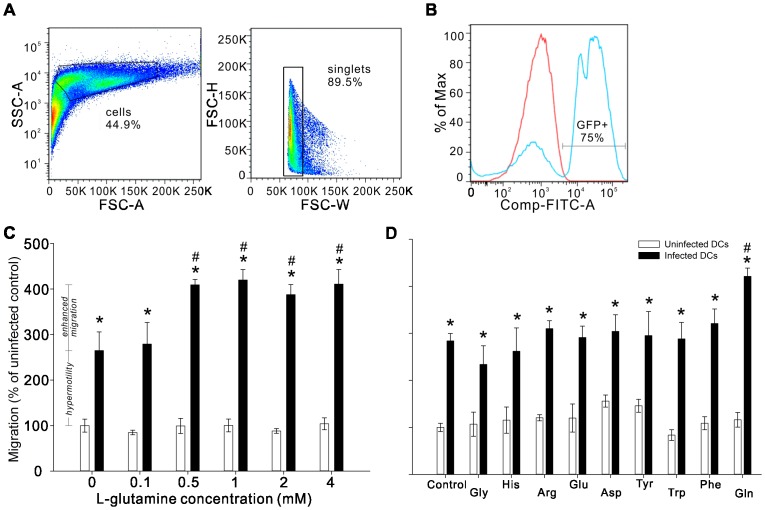
Transmigration assay of *T. gondii*-infected DCs *in vitro*. Infection frequency of *T. gondii*-infected DC culture was analyzed by flow cytometery. (A) In the gating strategy, SSC (side scatter) measures intracellular granularity and FSC (forward scatter) measures cell sizes. (B) The representative histogram shows the mean fluorescent intensity (x-axis) versus percentage of gated population (y-axis) for GFP. The pink histogram represents uninfected and the blue indicates infected DC culture. The infection frequency of DCs is about 75%. (C and D) DCs were infected with *T. gondii* for 4 hours and then cell migration was tested by Costar Transwell System. White bars represent uninfected DCs and black bars represent *T. gondii*-infected cells. Bar graphs depict mean values of migration ± SEM from three independent experiments performed in triplicate. Migration value is defined as the number of migrated cells in each condition normalized by the number of migrated cells in spontaneous migration, the migration of uninfected cells in the absence of factors (% of uninfected control). (C) “Hypermotility” state is defined as the increased migration after infection comparing to the spontaneous migration, the migration of uninfected control cells in the absence of factors. “Enhanced migration” to glutamine is the increased migration responding to increasing glutamine concentrations (between 0.5 and 4.0 mM) and is beyond that of the hypermotility induced by infection alone. Two-way ANOVA revealed an interaction effect between *T. gondii* infection and glutamine concentration (F_(5,37)_ = 4.426; P = 0.003). Further one-way ANOVA examining effects of glutamine within infected groups revealed an effect of glutamine in *T. gondii*-infected (P<0.01; indicated by asterisks) but not uninfected DCs. Post-hoc pairwise comparison of means for glutamine concentrations within the *T. gondii*-infected DCs revealed an increase in migration relative to control at 0.5, 1.0, 2.0, and 4.0 mM concentrations (Fisher's protected LSD, P<0.02; indicated by #), but not at 0.1 mM glutamine. (D) The specificity of enhanced migration to glutamine was tested by examining the cell migration in the presence of glutamine verse other amino acids such as glycine (Gly), histidine (His), arginine (Arg), glutamic acid (Glu), aspartic acid (Asp), tyrosine (Tyr), tryptophan (Trp) or phenylalanine (Phe). Two-way ANOVA revealed an interaction effect between *T. gondii* infection and amino acids (F_(9,70)_ = 2.765; P = 0.008). Further one way ANOVA examining effects of amino acids within infected groups revealed an effect of amino acids in *T. gondii*-infected (P<0.005; indicated by asterisks) but not uninfected DCs. Post-hoc pairwise comparison of means for amino acid condition within the infected DCs revealed an increase in migration relative to the control within only the increased glutamine condition (Fisher's protected LSD, P<0.001; indicated by #). The y-axis scale for both (C) and (D) is the same and shown at the far left.

We next used rat bone marrow-derived DC culture to study the effect of *T. gondii* infection on cell migration. Bone marrow-derived cells were isolated and cultured in complete medium supplemented with cytokines for seven days (see methods), and the phenotype of DCs was confirmed by flow cytometry (Fig. S2). DCs were infected by tachyzoites (multiplicity of infection, MOI, of 1) for 4 hours and then migration was examined by *in vitro* transwell system. Flow cytometric results show that the infection frequency of DCs is about 75% (Fig. 2A and 2B). *T. gondii* infection resulted in a roughly 2.5-fold increase in the number of DCs migrating to control medium, a result that we will refer to as a “hypermotility state” of infected DCs and one that has been reported previously [10] (Fig. 2C). Moreover, the infected cells exhibited an additional “enhanced migration” towards medium containing glutamine concentration above 0.5 mM (Fig. 2C). This enhanced migration was beyond that of the hypermotility induced by infection alone and was specific to glutamine relative to other amino acids, including the amino acids that utilize the same transporter (Fig. 2D). Importantly, uninfected DCs did not show induced migration to any of the tested glutamine concentrations or amino acids. This hypermotility and enhanced migration could not be explained simply by the maturation of the DCs because challenge with lipopolysaccharide (LPS) had no effect on migration to glutamine or other amino acids (Table S1).

### Enhanced migration of infected DCs towards glutamine is dependent on the glutamine transport via SNAT2 and availability of intracellular glutamine

**Figure 3:**
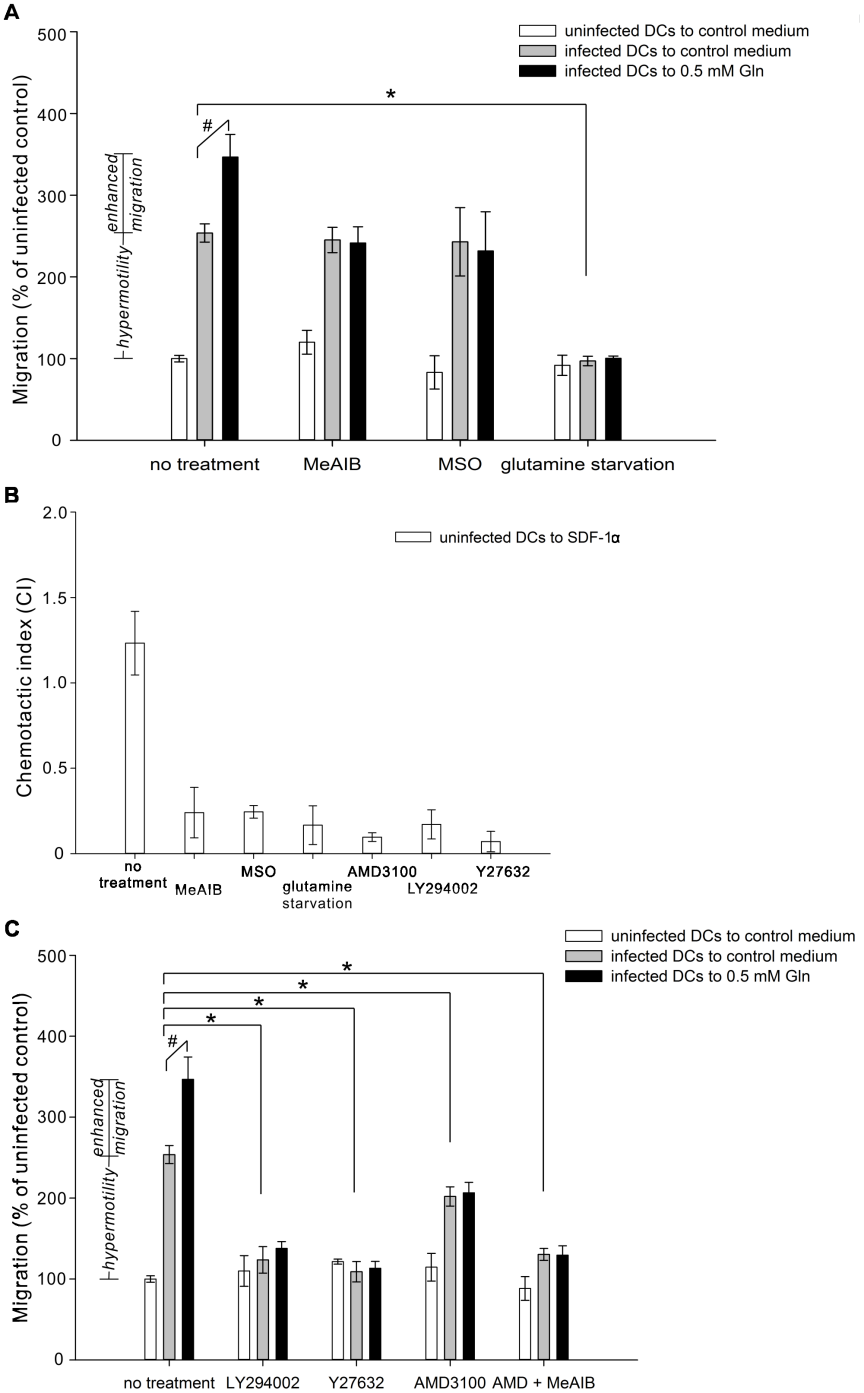
Pharmacological manipulation study of the induced migration of the *T. gondii*-infected and uninfected DCs. (A) Uninfected and infected DCs were left untreated (no treatment) or were treated with MeAIB (the competitive inhibitor of glutamine transport via SNAT2) or MSO (the inhibitor of glutamine synthetase), and cell migration to control medium (without glutamine) or to medium containing 0.5 mM glutamine was studied. A pretreatment starving the DCs of glutamine was also used to examine the importance of glutamine source in hypermotility and enhanced migration. (B) Uninfected control DCs were treated with MeAIB, MSO, inhibitors of CXCR4 (AMD3100), PI3K (LY294002) or Rho kinase (Y27632), or Gln starvation for 2 hours before assessing migration to 100 ng/ml SDF-1α. Chemotactic index (CI) is defined as the fold increase in the number of migrating DCs to SDF-1α over the spontaneous migration. One-way ANOVA reveals an effect of pharmacological treatments on the SDF-1α-induced migration (F_(6,44)_ = 6.700, P<0.001). Asterisks indicate P<0.05 (Dunnett's post hoc). (C) LY294002, Y27632, AMD3100 and the combination of AMD3100 and MeAIB were used to study the potential mechanisms contributed to the hypermotility and the enhanced migration of *T. gondii*-infected DCs. White bars represent uninfected DCs, and gray and black bars represent infected cells in response to 0 mM and 0.5 mM glutamine, respectively. Bar graphs depict mean values of migration ± SEM from three independent experiments performed in triplicate. “No treatment” bars in (A) and (C) represent the same data. One way ANOVA examining effects of treatment across the uninfected control DCs revealed no effect of treatment on cell migration (P>0.5). One-way ANOVA examining effects of pharmacological treatments on hypermotility within infected groups (gray bars in A and C) revealed an effect of pharmacological treatments in *T. gondii*-infected DCs (F_(7,49)_ = 16.541, P<0.001). Asterisks indicate P<0.003 (Fisher's protected LSD). Within each pharmacological treatment, # indicates significant effects of 0.5 mM glutamine (enhanced migration to glutamine) relative to control conditions (hypermotility) (Student's t-test, P<0.01).

A glutamine analog (2-(methylamino)-isobutyrate, MeAIB), which is a specific substrate of SNAT2 and decreases the cellular uptake of glutamine [25], [26], was able to block the enhanced migration to 0.5 mM glutamine but had no effect on the hypermotility of infected DCs (Fig. 3A). A glutamine synthetase inhibitor (methionine sulfoximine, MSO) also blocked the enhanced migration to glutamine but not the hypermotility. Extracellular glutamine was always present in the media during the experiment (see methods), and thus, these results suggest that glutamine uptake by SNAT2 and glutamine synthetase are both required for the enhanced migration. In contrast, complete glutamine starvation of the infected DCs for 2 hours prior to the assay abolished both enhanced migration to glutamine and hypermotility, indicating that glutamine resource is essential for both hypermotility and enhanced migration of infected DCs.

### Glutamine transport via SNAT2 is important for CXCR4-dependent migration of uninfected DCs to SDF-1α

In order to test if SNAT2 serves as a general factor influencing immune cell migration, we examined another scenario in which uninfected DCs migrate, in this case to SDF-1α, a migration that has been shown to be dependent on CXCR4 signaling. Examining SDF-1α-induced transmigration in uninfected DCs with the same treatments described above, we demonstrated that glutamine starvation, MeAIB or MSO significantly reduced the uninfected DC migration to SDF-1α (Fig. 3B). Both a CXCR4 antagonist (AMD3100) and a PI3K inhibitor (LY294002) have previously been shown to block the SDF-1α-induced migration in bone marrow-derived mesenchymal stem cells, T lymphocytes and platelets [27]–[30]. We demonstrated that either of these two drugs, as well as the specific Rho kinase inhibitor (Y27632), also dramatically decreased the migratory ability of uninfected DCs to SDF-1α (Fig. 3B). Thus, SDF-1α-induced migration of uninfected DCs is dependent on SNAT2 transport of glutamine, functional glutamine synthetase as well as the CXCR4-PI3K-Rho kinase pathways.

### Both hypermotility and enhanced migration to glutamine of infected DCs are dependent on the CXCR4- PI3K-Rho kinase pathways

We then tested if these CXCR4-PI3K-Rho kinase-related inhibitors could affect hypermotility and enhanced migration of *T. gondii*-infected DCs in the presence of glutamine. After 2 hours of *T. gondii* infection, DCs were treated for an additional 2 hours with the same concentration of these inhibitors of the CXCR4-PI3K-Rho kinase pathways described previously. Inhibiting PI3K or Rho kinase blocked both the hypermotility and the enhanced migration to glutamine in infected cells (Fig. 3C). The CXCR4 inhibitor, AMD3100, inhibited the enhanced migration to glutamine and partially decreased the hypermotility, which suggested that CXCR4 is not the only activator of the downstream PI3K pathway that contributes to the migratory state of infected cells. In order to determine if glutamine transport through SNAT2 could be coactivating this pathway and thereby contributing to migration, we used the combination of MeAIB and AMD3100 to completely block enhanced migration and reduced hypermotility to close to the control level. The viability of intracellular *T. gondii* following these pharmacological treatments was measured by plaque assays, and none of the inhibitors affected virulence of the parasites (Fig. S3), indicating that the decrease of induced migration of infected DCs was not due to the loss of parasite viability or stalled replication. Based on these results, we developed a hypothesis that hypermotility and enhanced migration of *T. gondii*-infected DCs may be dependent on an interaction between CXCR4 and glutamine transport via SNAT2 in activating the PI3K-Rho kinase pathways.

### *T. gondii* infection activates DCs and the SNAT2 and CXCR4 expression in the host cell remains unchanged

**Figure 4:**
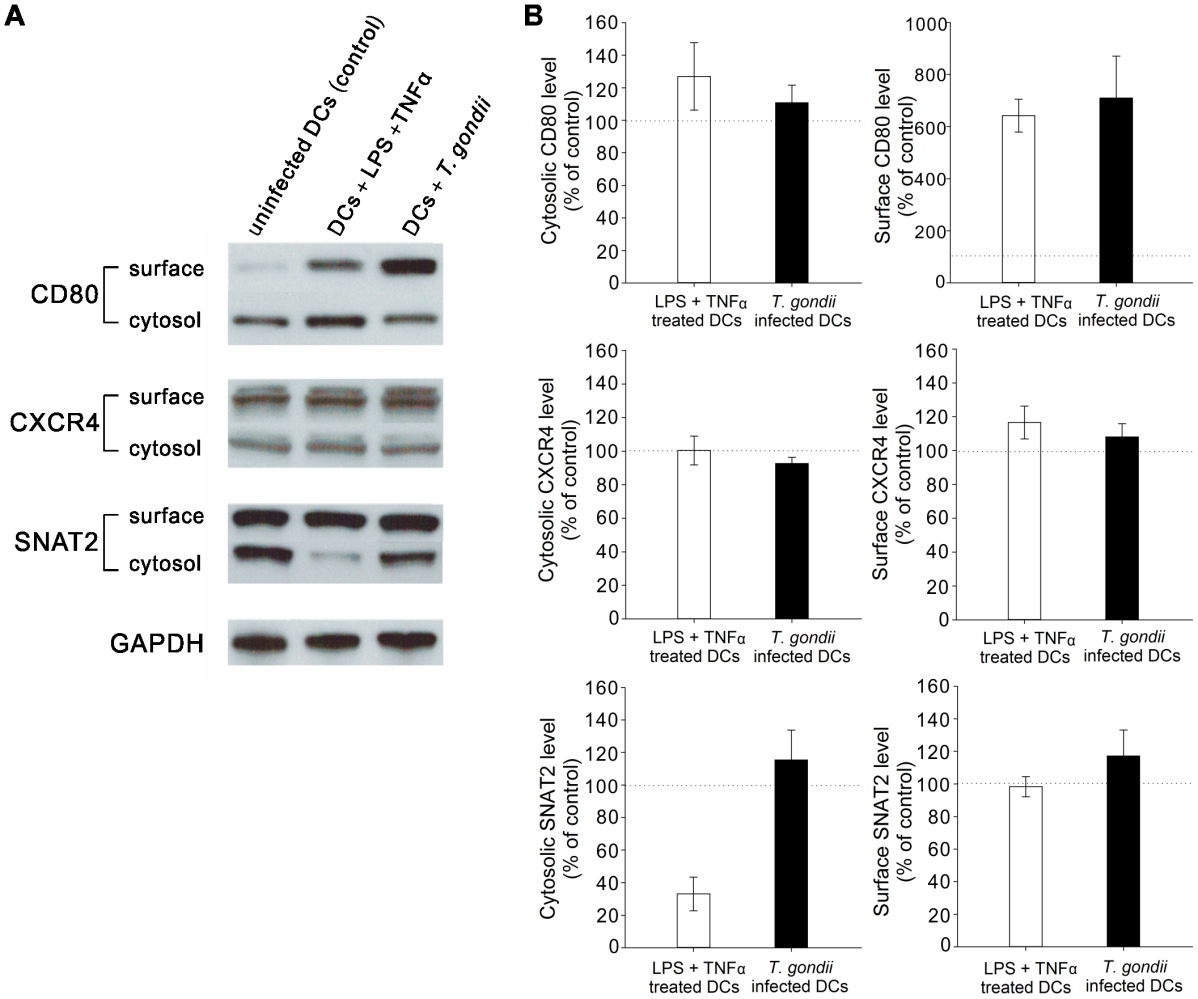
Western blot analysis of CXCR4 and SNAT2 expression of *T. gondii*-infected DCs *in vitro*. (A) DCs were treated with 100 ng/ml LPS+50 ng/ml TNFα or were infected with *T. gondii* for 2 hours in the presence of 2 mM glutamine and then the protein expression of CD80, CXCR4 and SNAT2 in cytosol and on the surface were studied. GAPDH is the loading control. (B) The band intensity in the blots was quantified to show the relative protein level of CD80, CXCR4 and SNAT2 in tested groups compared to control (dotted line). Experiments were repeated three times and quantified by Image J.

To determine whether *T. gondii* affected CXCR4 and SNAT2 protein expression in host cells, we used Western blot to compare the protein expression on surface and in cytosol in DCs either exposed to live *T. gondii* or an alternative immune activator (LPS + tumor necrosis factor α, TNFα). We also examined CD80 protein expression as a marker of immune cell activation/maturity. Figure 4A shows that both LPS + TNFα treatment and *T. gondii* infection increased CD80 protein expression on the surface of DCs, which suggests that both stimuli result in cell activation and maturation. The quantification of the density of the bands in the blots shows that although cell maturation with either LPS + TNFα or *T. gondii* infection did not affect either surface or cytosolic CXCR4 expression or surface SNAT2 expression, LPS + TNFα treatment decreased SNAT2 in cytosol while *T. gondii*-infected DCs possessed similar levels as uninfected DCs (Fig.4B).

## Discussion

*T. gondii* is an obligate intracellular pathogen that acquires glutamine from the host to carry out functions necessary for its metabolic activity, invasion, and replication. Here, we report that infection, replication and viability in *T. gondii* are greatly facilitated by glutamine availability. We demonstrate that in infected immune cells (DCs) *in vitro*, *T. gondii* induces a) a hypermotility state and, b) a specific enhanced migration to environments with high glutamine levels (≧0.5 mM). This hypermotility and enhanced migration are dependent on SNAT2 transport of glutamine, the presence of functional glutamine synthetase and the CXCR4-PI3K-Rho kinase pathways. Whereas activation with LPS + TNFα results in downregulation of SNAT2 protein expression in DCs, this expression is unchanged with *T. gondii* infection and may contribute to the induced migration of *T. gondii*-infected cells. These findings support the hypothesis that *T. gondii* manipulates the migratory properties of the host cell to increase the probability of migrating to places with sufficient glutamine resource to meet its metabolic requirements.

The glutamine dependency of *T. gondii* is well documented. The Toxoplasma TCA cycle, which utilizes glucose and glutamine, generates energy for intracellular growth and replication; inhibition of the cycle leads to the death of parasites [2], [4]. However, mutation of the *T. gondii* glucose transporter only has a minor effect on the growth of the parasite, and glutamine, but not glucose, is indispensable to its survival [3]. Therefore, glutaminolysis is essential for the parasitic requirements of *T. gondii*. Recently a GABA shunt was observed for regulating the catabolism of glutamine/glutamate in the Toxoplasma TCA cycle [2], and DCs infected with live *T. gondii* have been demonstrated to secrete GABA and exhibit hypermotility; this induced migration may increase the chance of wide dissemination of the parasites during acute infection [10], [31], [32]. Glutamine serves as the precursor of GABA; thus it is plausible that the rapidly dividing *T. gondii* imposes a high glutamine burden on the host, which may manipulate the migratory phenotype of the infected immune cells in order to sustain intracellular glutamine concentrations. Our results support this idea as infected DCs exhibit the hypermotility phenotype and enhanced migration to glutamine (Fig. 2). On the other hand, although glutamine-induced migration of *T. gondii*-infected DCs is a new finding, it is not the first amino acid that was reported to be a chemotaxis-inducing factor for immune cells, as glutamate has been shown to attract neutrophils to migrate to the inflamed/wound tissue via activating the downstream PI3K pathway [33], [34].

The hypermotility of *T. gondii*-infected DCs is related to the parasite strain, and type II strain, as used in this study, exhibits the strongest induction of hypermotility [11]. This hypermotility has been suggested to be independent of CCR5, CCR7 or Toll/interleukin-1 pathways but to involve a G*i* protein signaling transduction [10], [32]. Chemokine-receptor complexes are known to initiate signal transduction events leading to cellular responses such as leukocyte chemotaxis and adhesion. CXCR4, one of 19 known chemokine receptors in mammals, couples primarily through G*i* proteins, and PI3K and Rho kinase pathways involved in cell motility and orientation are downstream to activation of CXCR4 [12], [13], [35], [36]. While we also demonstrate the crucial roles of PI3K and Rho kinase in this migration, as inhibition of either pathway blocked enhanced migration to glutamine and drastically reduced the hypermotility in *T. gondii*-infected DCs, AMD3100, an antagonist that binds CXCR4 without engaging receptor signaling [37], [38], blocked enhanced migration to glutamine but only partially decreased hypermotility. On the other hand, the combination of AMD3100 and MeAIB, a specific SNAT2 inhibitor, completely blocked enhanced migration and significantly reduced hypermotility, indicating that CXCR4 and SNAT2 may cumulatively regulate the altered migratory phenotype (Fig. 3). While the exact role of CXCR4 and SNAT2 in the induced migration of infected DCs is unclear, CXCR4 promotes the activation of PI3K pathway, and SNAT2, which has been suggested to possess a dual transporter/receptor (transceptor) function in cultured muscle cells and fibroblasts, activates PI3K pathway and regulates/senses changes of the intracellular amino acid pool and the availability of extracellular amino acids [14], [15], [39], [40]. The transmigration results in Figure 3 support an interaction between SNAT2 and CXCR4 pathways through which *T. gondii* may orient increased migration of infected DCs to environments with high glutamine level.

In this study, western blot analysis showed that whereas DCs challenged with LPS + TNFα downregulate cytosolic SNAT2 protein, *T. gondii*-infected DCs possessed a similar level of the cytosolic pool of SNAT2 protein as uninfected control cells. Downregulation of SNAT2 following LPS + TNFα challenge is, to our knowledge, a novel finding and further study will be required to understand the role of SNAT2 in activated immune cells. It has been shown that during the acute phase of amino acid deprivation, the adaptive regulation of SNAT2 activity relies on recruitment of preformed proteins from a cytosolic pool to the cell membrane [41]–[43], which may explain why *T. gondii*-infected cells with unchanged SNAT2 expression in cytosol and on cell surface possessed the altered migratory phenotype, whereas LPS-treated immune cells did not (Table S1). CXCR4 protein remains unchanged with both LPS + TNFα treatment and *T. gondii* infection, indicating that expression of CXCR4, along with SNAT2, may be permissive for the migratory phenotype even if expression does not increase with enhanced migration.

**Figure 5:**
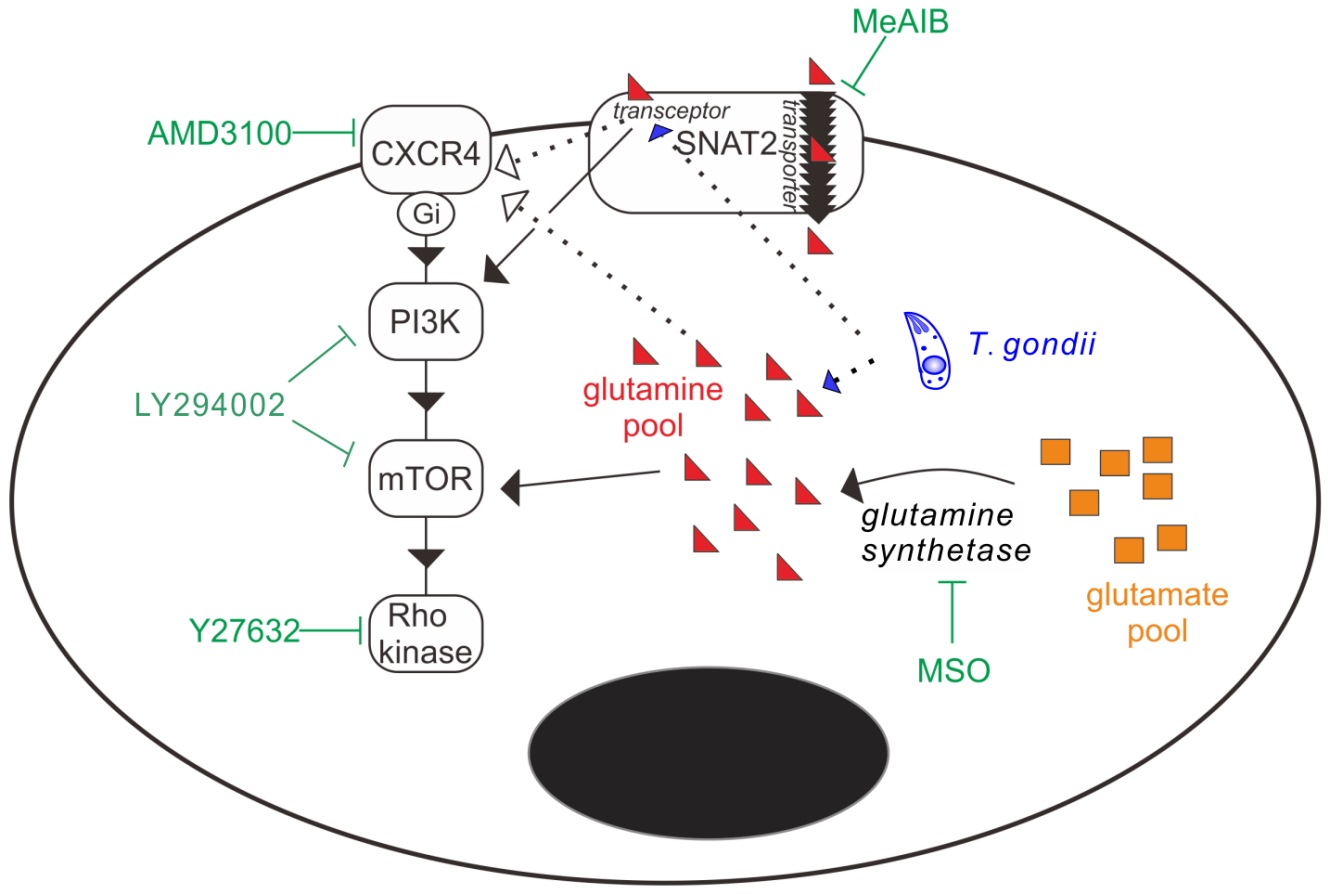
Schematic diagram of the hypothetical model. *T. gondii* increases the likelihood that infected immune cells will migrate to glutamine-rich environment by “turning on” the transceptor function of SNAT2 and modulating the CXCR4-PI3K-Rho kinase signaling pathways which are known to regulate migration. Dotted lines denote the new possible interactions between *T. gondii*, SNAT2 and CXCR4.

Based on these studies, it is unclear if the enhanced migration to glutamine is caused by *T. gondii* directly or by the host cell trying to rebalance the glutamine homeostasis. New evidence also shows that LY294002 targets mammalian target of rapamycin (mTOR) at the same concentrations that inhibit PI3K, and mTOR is involved in amino acid-sensing pathways [44]–[46]. However, based on our results, a new hypothetical mechanism of how *T. gondii* induces migration in infected DCs was proposed (Fig. 5). Firstly, a new on-and-off theory about the transceptor role of SNAT2 was proposed in 2009, and SNAT2 is thought to be “closed off” when sufficient concentrations of its substrates exist in the intracellular compartment, and to be activated (“on” state) when intracellular amino acid concentrations drop [47]. The off state of the transceptor might explain why immature DCs expressing SNAT2 do not show hypermotility or enhanced migration to glutamine in this study. We hypothesized that *T. gondii* may “turn on” the transceptor function of SNAT2. A drop in intracellular glutamine concentration secondary to parasite infection may be sufficient to turn on SNAT2; however, we cannot rule out the potential influence of *T. gondii*-related proteins. One of the ways in which *T. gondii* has been shown to manipulate host cell function is by secretion of rhoptry proteins or dense granule proteins into host cell cytoplasm and nucleus during invasion [48], [49]; dense granule protein GRA5 has been shown to trigger the migration of human dendritic cells toward CCL19 [50], and rhoptry proteins and dense granule proteins of *T. gondii* interfere with host signaling pathways including immunity-related GTPases (IRGs) and nuclear factor-κB (NF-κB) pathways [49], [51], [52]. Therefore, *T. gondii* might directly or indirectly turn on the transceptor role of SNAT2 by modulating the intracellular glutamine metabolism and/or secreting modulatory protein(s) in the infected immune cells.

In summary, we hypothesize that *T. gondii* metabolic requirements may lead to parasite-host interactions that benefit the parasite by increasing the probability that infected-immune cells will carry the parasite out of circulation into tissues with sufficient glutamine (e.g. brain, eye, muscle, lung…). Technical challenges moving forward include measuring intracellular concentration of radioactive labeled glutamine in *T. gondii*-infected DCs, delivering RNAi targeting CXCR4 and/or SNAT2 to *T. gondii*-infected immune cells without altering *T. gondii* or immune cell viability, manipulating the extracellular glutamine concentration *in vivo* to redirect the migration of infected cells, and the lack of knockout animal models of CXCR4 or SNAT2 to study the dissemination of the parasites. We here provide evidence for the CXCR4 and SNAT2 pathways as novel mechanisms for immune cell migration and potential targets for prevention of parasite dissemination and chronic infection in rodents and humans.

## Materials and Methods

### Parasites

Prugniaud stain of *T. gondii* expressing firefly luciferase and green fluorescent protein (GFP) was a gift from Dr. John Boothroyd. Tachyzoites were maintained by serial 2-day passage in human foreskin fibroblast (HFF) monolayers cultured in complete medium (DMEM plus 10% fetal bovine serum, 1% penicillin-streptomycin, and 2 mM L-glutamine) (Life Technology).

### Animals

Pregnant female Sprague-Dawley rats (single caged; Charles River Laboratories) were used for generation of pup bone marrow derived immune cells. All procedures were reviewed and approved by the Stanford University Administrative Panel on Laboratory Care and the Association for Assessment of Laboratory Animal Care.

### *T. gondii* Invasion Assay

HFFs were cultured on 12 mm coverslips in complete medium 24 hours before the experiment. Tachyzoites were added to HFFs (MOI = 2) and invasion in the absence (0 mM) or presence (2 mM) of glutamine was allowed for 6 hours at 37°C in the incubator with 5% CO_2_ and then washed by PBS three times and stained with anti-SAG1 (1∶10,000) for 15 minutes, which labels extracellular parasites before 4% paraformaldehyde fixation. Coverslips were then blocked for 30 minutes and incubated in Alexa Fluor 555 (1∶1000) for 2 hours. A Zeiss confocal laser scanning microscope (LSM) with a 40× objective was used to count the number of parasites and total of 15 fields per coverslip were examined. The ratio of intracellular tachyzoites to total tachyzoites in the examined field on the coverslip is defined as infection efficiency.

### *T. gondii* Replication Ability Assay

HFF culture conditions were identical to the invasion assay. HFFs were challenged with tachyzoites (MOI = 1) in complete medium (which contains 2 mM L-glutamine) for 6 hours and then washed with PBS to remove glutamine-rich medium and extracellular tachyzoites before incubating in media with or without 2 mM L-glutamine for another 16 hours. SAG1 antibody was used to label extracellular parasites and stained with Alexa Fluor 555 for 2 hours. The number of replication cycles undergone by intracellular *T. gondii* was quantified for 15 fields under a Zeiss confocal LSM with a 63× objective, and the mean replication cycle was presented as the replication ability. No parasites underwent more than 4 rounds of replication cycle in the time period that we chose.

### *T. gondii* Viability Assay

Free tachyzoites were incubated in the medium containing 0 or 2 mM of L-glutamine or 2 mM of D-glutamine for 4 hours at 37°C in the incubator with 5% CO_2_, and then yellow MTT (which is reduced to purple formazan by mitochondrial enzymes) was added in living cells and the absorbance of colored solution was quantified by a spectrophotometer.

### Bone Marrow-Derived Dendritic Cell Culture

The bone barrow-derived DC culture was generated as previously described [53], with slight modifications. Briefly, bone marrow from 16 day-old Sprague-Dawley rat pups was isolated and stored in liquid nitrogen until use. Upon thawing, cells were washed with RPMI 1640 (Catalog number 21870, Life Technology) and placed in T-75 flasks at a density of approximately 2.5-3.5×10^6^ cells/ml in complete medium: RPMI 1640, 10% fetal bovine serum, 2 mM L-glutamine, 1% nonessential amino acids, 1 mM sodium pyruvate, 50 units/ml penicillin +50 ug/ml streptomycin (all from Life Technology) and the cytokines, interleukin-4, granulocyte-macrophage colony stimulating factor (GM-CSF), Flt-3 ligand (5 ng/ml per cytokine, R&D Systems). On the third day, each flask was given 5 ml of fresh complete medium containing GM-CSF only, and then three quarters of medium containing non-adherent cells were replaced with fresh complete medium with GM-CSF on day 5. Cells were used on day 7.

### Flow Cytometry

DCs were infected with freshly egressed *T. gondii* (MOI = 1) for 4 hours and then collected and washed in FACS buffer (1 mM EDTA+1% FBS in PBS) on Day 7. An LSR II flow cytometer (BD Biosciences) was used to investigate the infection frequency and the results were analyzed with FlowJo Software (Tree Star, Inc.).

### *In Vitro* Transmigration Assay

Cell migration was tested using Transwell assays (Costar 24-well plate with inserts, 5 µm pore). Briefly, 1 million cells challenged with freshly egressed *T. gondii* (MOI = 1) for 4 hours were resuspended in culture medium consisting of RPMI 1640 with 1% bovine serum albumin (BSA) and 2 mM L-glutamine in a 100-µl volume. Another group of cells were infected with *T. gondii* the same way for 2 hours following another 2 hours of inhibitor treatments before being resuspended in culture medium as described above. Infected and inhibitor-treated cells were then counted by trypan blue staining to confirm the cell viability before being transferred to the transwell system. Tested amino acids or SDF-1α (final working concentration: 100 ng/ml) diluted in the control medium (RPMI 1640+1% BSA) were added to the bottom well in a 600-µl volume when the same amount of live cells in each condition were added to the upper wells in a 100-µl volume. The migration was assayed for 1.5 hours in a 37°C incubator with 5% CO_2_ and then migrated cells were collected and counted by trypan blue staining with a hemocytometer.

The tested amino acids were diluted in control medium at the concentrations as following: 0.133 mM glycine, 0.0968 mM histidine, 1.15 mM arginine, 0.136 mM glutamic acid, 0.15 mM aspartic acid, 0.129 mM tyrosine, 0.0245 mM tryptophan, 0.0909 mM phenylalanine (Sigma). Because the formula of RPMI 1640 contains a mixture of amino acids with the concentrations described above, adding another equal amount of amino acids in the migration medium created 2× of tested amino acids, which were comparable to 4 mM of glutamine (2× Gln). Inhibitors were used at following final concentrations: 40 mM MeAIB (Sigma), 5 mM MSO (Sigma), 50 µM LY294002 (Cell Signaling), 10 µM Y27632 (Sigma), 50 µM AMD3100 (Abcam).

### Cell Surface Protein Biotinylation and Western Blot

Surface proteins of DCs were isolated by using the Pierce Cell Surface Protein Isolation kit (Thermo Scientific) according to the manufacturer's protocol. In brief, cells grown in Falcon T-75 flasks were washed with PBS, incubated with EZ-LINK Sulfo-NHS-SSbiotin for 30 minutes at 4°C and then lysed with lysis buffer containing protease inhibitor. The biotinylated surface proteins were trapped within NeutrAvidin agarose gel when cytosol proteins were eluted, and then surface proteins were eluted by Bio-Rad sample buffer containing DTT.

Protein samples (25 µg) were separated on 10% SDS-PAGE gel and then transferred to Bio-Rad PVDF membranes and immunoblotted with antibodies against SNAT2 (1∶500, Santa Cruz biotechnology), CXCR4 (1∶500, Abcam) and CD80 (1∶10000, Abcam). Glyceraldehyde 3-phosphate dehydrogenase (GAPDH) was used as a loading control (1∶10000, Sigma). The density of bands on 3 independent blots was quantified by Image J.

### Statistical Analysis

Statistical analyses were performed using IBM SPSS statistics software (version 20).

## Supporting Information

Figure S1 **Viability assay of** ***T. gondii*** **-infected HFFs** ***in vitro*** **.**
(DOCX)

Figure S2 **Characterization of rat bone marrow-derived DC cultures on Day 7.**
(DOCX)

Figure S3 **Viability assay of intracellular** ***T. gondii*** **in HFFs following inhibitor treatments.**
(DOCX)

Table S1 **Transmigration assay *in vitro*: cell migration of activated DCs to glutamine or other tested amino acids.**
(DOCX)
