# Supplementary material for: Toxoplasma gondii Is Dependent on Glutamine and Alters Migratory Profile of Infected Host Bone Marrow Derived Immune Cells through SNAT2 and CXCR4 Pathways
Source: PLoS One. 2014 Oct 9;9(10):e109803. doi: 10.1371/journal.pone.0109803 (PMC4192591; doi:10.1371/journal.pone.0109803)
Supplement: Figure S1 — Viability assay of T. gondii -infected HFFs in vitro . (DOCX) [file pone.0109803.s001.docx]

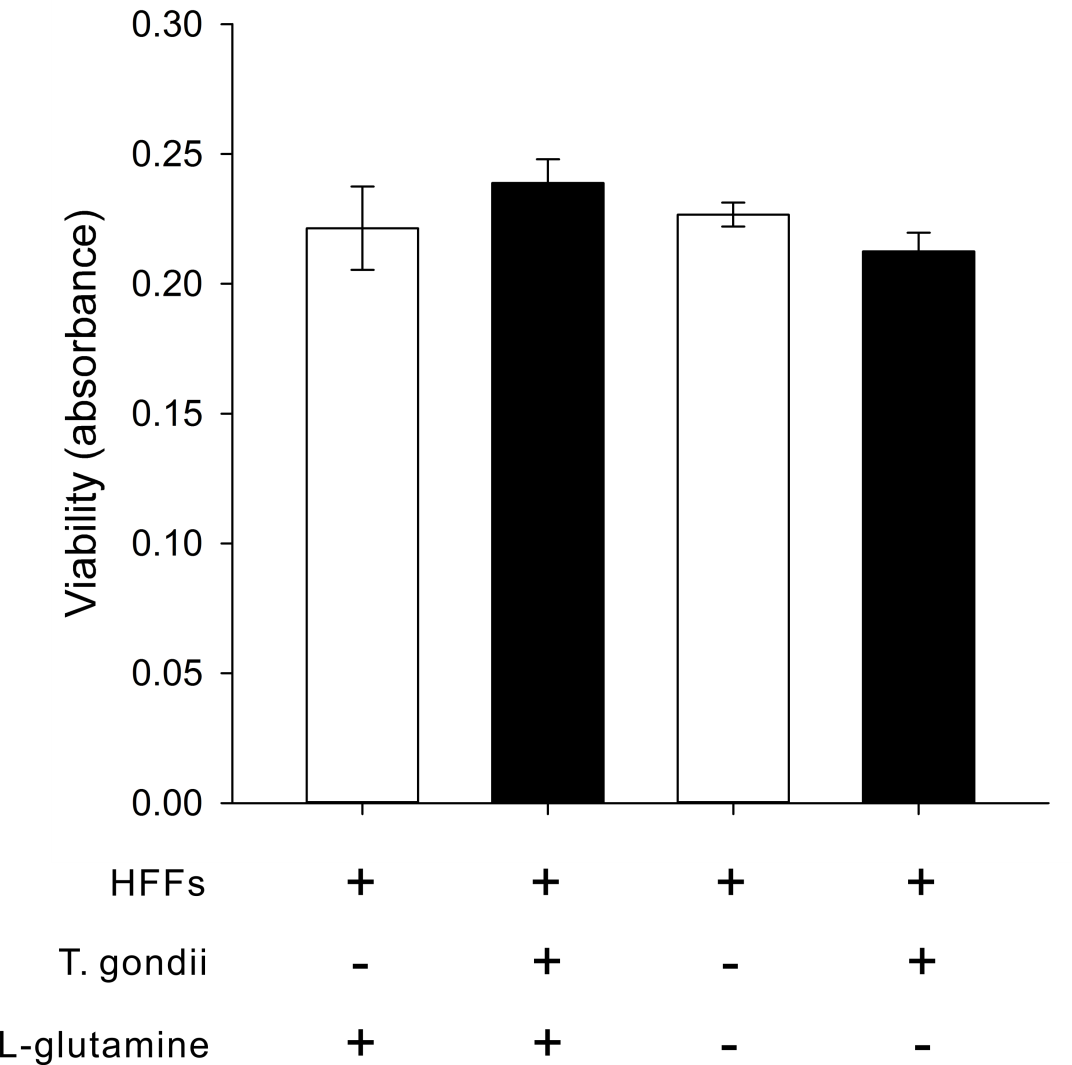


**Figure S1. Viability assay of *T. gondii*-infected HFFs *in vitro*.** Confluent HFF monolayer in 96-well plates was infected with *T. gondii* in the presence of L-glutamine for 6 hours. Extracellular parasites were then washed away with 1X PBS, and the fresh medium containing 0mM or 2mM of L-glutamine was added to the cells. 24 hours later, the viability of infected (black bars) and uninfected (white bars) HFFs was examined by MTT assay. Bar graphs depict mean values of absorbance ± SEM from three independent experiments performed in decuple. No significant differences were found between conditions (p > 0.5, one-way ANOVA).
