# Supplementary material for: Toxoplasma gondii Is Dependent on Glutamine and Alters Migratory Profile of Infected Host Bone Marrow Derived Immune Cells through SNAT2 and CXCR4 Pathways
Source: PLoS One. 2014 Oct 9;9(10):e109803. doi: 10.1371/journal.pone.0109803 (PMC4192591; doi:10.1371/journal.pone.0109803)
Supplement: Figure S2 — Characterization of rat bone marrow-derived DC cultures on Day 7. (DOCX) [file pone.0109803.s002.docx]

**
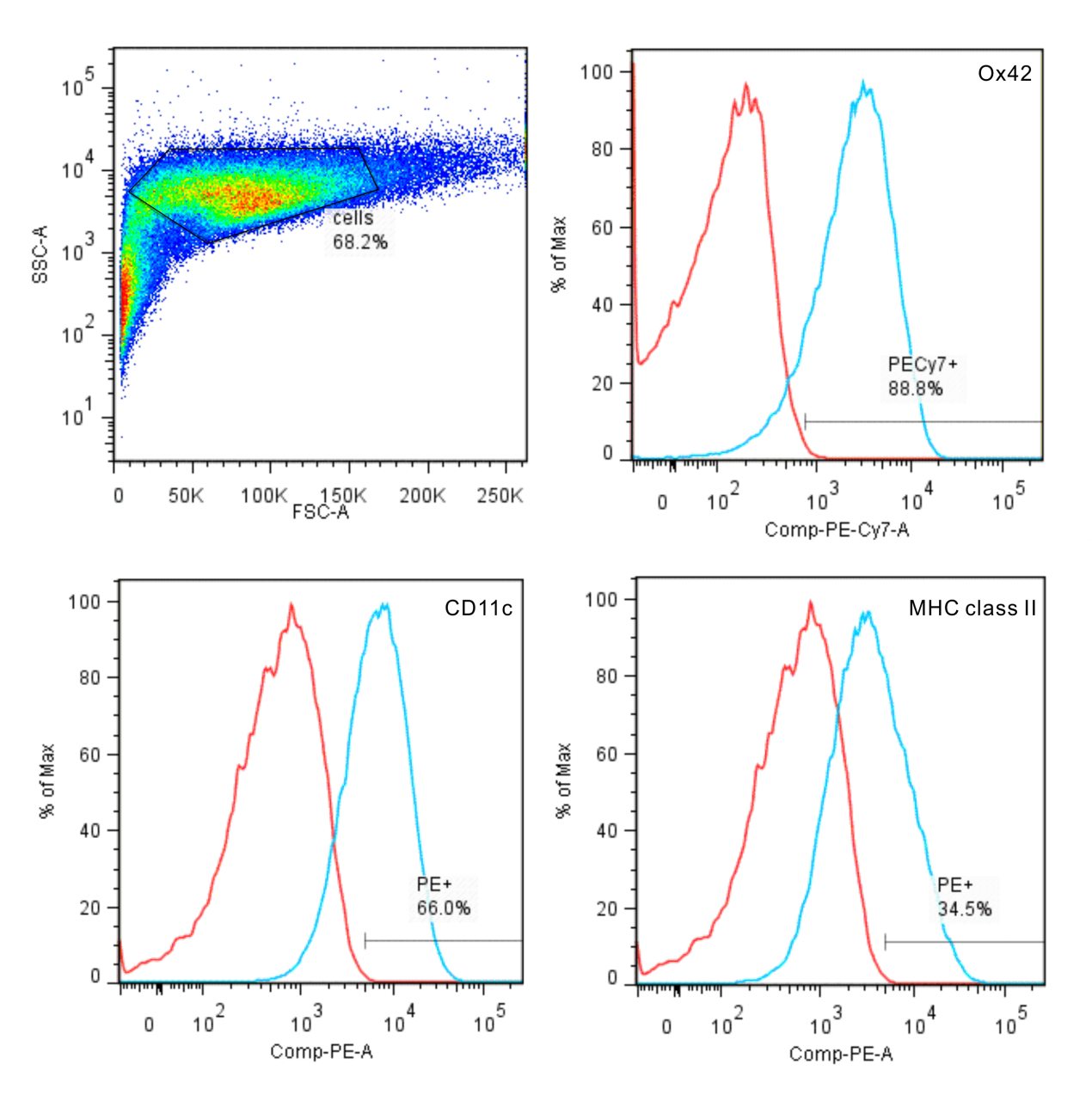
**

**Figure S2. Characterization of rat bone marrow-derived DC cultures on Day 7.** Flow cytometric analysis of DC cultures showing forward- and side-scatter dot plot with the gated population prior to singlet gating (upper left panel), and histograms of mean fluorescent intensity (x-axis) versus percentage of gated population (yaxis) for OX42, CD11c, MHC class II. 88.8%, 66% and 34.5% of the cells expressed these markers (blue histograms), respectively. The pink histograms represent the background staining of isotype-matched control antibodies. Results shown are representative of two independent experiments, assaying 20,000 cells per experiment.
