# Supplementary material for: Toxoplasma gondii Is Dependent on Glutamine and Alters Migratory Profile of Infected Host Bone Marrow Derived Immune Cells through SNAT2 and CXCR4 Pathways
Source: PLoS One. 2014 Oct 9;9(10):e109803. doi: 10.1371/journal.pone.0109803 (PMC4192591; doi:10.1371/journal.pone.0109803)
Supplement: Figure S3 — Viability assay of intracellular T. gondii in HFFs following inhibitor treatments. (DOCX) [file pone.0109803.s003.docx]

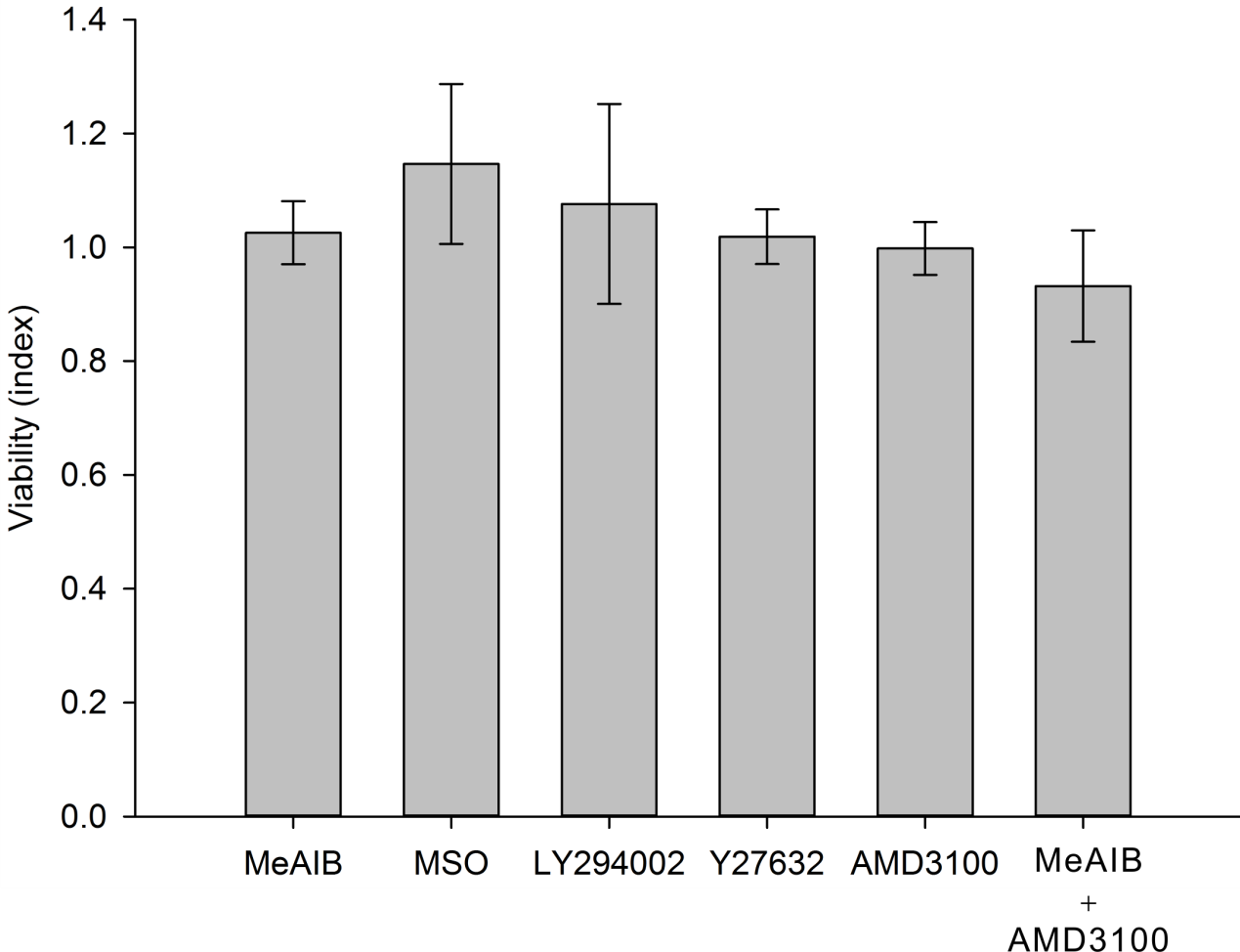


**Figure S3. Viability assay of intracellular *T. gondii* in HFFs following inhibitor treatments.** Intracellular parasites were treated with inhibitors for 2 hours, syringe-lysed, centrifuged and then resuspended in fresh complete medium. 500 parasites were added to 6-well plates and grown undisturbed for at least 6 days. The number of plaques, indicating individual viable parasite, was counted on Day 7 and relative viability of T. gondii after treatments is shown. Viability index is defined as the number of plaques in each treated culture normalized by the number in untreated cultures. Bar graphs depict mean values of viability index ± SEM from two independent experiments. No significant differences were found between conditions (p > 0.5, one-way ANOVA).
