# Supplementary material for: Toxoplasma gondii Is Dependent on Glutamine and Alters Migratory Profile of Infected Host Bone Marrow Derived Immune Cells through SNAT2 and CXCR4 Pathways
Source: PLoS One. 2014 Oct 9;9(10):e109803. doi: 10.1371/journal.pone.0109803 (PMC4192591; doi:10.1371/journal.pone.0109803)
Supplement: Table S1 — Transmigration assay in vitro: cell migration of activated DCs to glutamine or other tested amino acids. (DOCX) [file pone.0109803.s004.docx]

**Table S1.** **Transmigration assay *in vitro*: cell migration of activated DCs to glutamine or other tested amino acids.**

| **1 μg/ml LPS** | **Tested medium** | **Migration value** | **SEM** |
| --- | --- | --- | --- |
| No treatment | Control medium (0 mM Gln) | 1 | ± 0.1 |
| Overnight treatment | Control medium (0 mM Gln) | 0.78 | ± 0.06 |
| Overnight treatment | 0.1 mM Gln | 0.89 | ± 0.27 |
| Overnight treatment | 0.5 mM Gln | 0.71 | ± 0.16 |
| Overnight treatment | 2× Gly | 0.66 | ± 0.02 |
| Overnight treatment | 2× His | 0.79 | ± 0.003 |
| Overnight treatment | 2× Arg | 0.69 | ± 0.09 |
| Overnight treatment | 2× Glu | 0.86 | ± 0.05 |

Mean values of migration ± SEM from three independent experiments performed in triplicate are shown. The number of migrating LPS-acitivated DCs (overnight treatment) in the presence of each test factor is normalized by the spontaneous migration of uninfected DCs (no treatment) in the absence of test factors (the control medium).
